# Don’t Lose Your Brain at Work – The Role of Recurrent Novelty at Work in Cognitive and Brain Aging

**DOI:** 10.3389/fpsyg.2017.00117

**Published:** 2017-02-06

**Authors:** Jan Oltmanns, Ben Godde, Axel H. Winneke, Götz Richter, Claudia Niemann, Claudia Voelcker-Rehage, Klaus Schömann, Ursula M. Staudinger

**Affiliations:** ^1^Human Resources Management, Daimler AGBremen, Germany; ^2^Psychology and Methods, Jacobs University BremenBremen, Germany; ^3^Project Group Hearing, Speech and Audio Technology, Fraunhofer Institute for Digital Media TechnologyOldenburg, Germany; ^4^Federal Institute for Occupational Safety and HealthDortmund, Germany; ^5^Institute of Applied Movement Science, Chemnitz University of TechnologyChemnitz, Germany; ^6^German Institute for Adult Education, Leibniz Centre for Lifelong LearningBonn, Germany; ^7^Robert N. Butler Columbia Aging Center, Columbia University, New YorkNY, USA

**Keywords:** plasticity, lifespan development, cognitive aging, use it or lose it, gray matter volume, work-task changes, job complexity, healthy aging at work

## Abstract

Cognitive and brain aging is strongly influenced by everyday settings such as work demands. Long-term exposure to low job complexity, for instance, has detrimental effects on cognitive functioning and regional gray matter (GM) volume. Brain and cognition, however, are also characterized by plasticity. We postulate that the experience of novelty (at work) is one important trigger of plasticity. We investigated the cumulative effect of recurrent exposure to work-task changes (WTC) at low levels of job complexity on GM volume and cognitive functioning of middle-aged production workers across a time window of 17 years. In a case-control study, we found that amount of WTC was associated with better processing speed and working memory as well as with more GM volume in brain regions that have been associated with learning and that show pronounced age-related decline. Recurrent novelty at work may serve as an ‘*in vivo*’ intervention that helps counteracting debilitating long-term effects of low job complexity.

## Introduction

Occupational health psychology aims to support the creation of “healthy workplaces in which people may produce, serve, grow, and be valued” (Quick et al., 1997, p. 3). It is surprising, however, that “age” or “aging” which have strong associations with physical and cognitive health (see Schaie, 2005; Baltes et al., 2006; Dekkers-Sánchez et al., 2008; Lidwall et al., 2009), have received less attention in organizational health research until now (Oltmanns et al., 2016). More often than not, chronological age has merely been used as a covariate or confound (De Lange et al., 2006; Schalk et al., 2010). Thus far, cognitive and brain health have been neglected as focal points in occupational health research and rarely are they studied across time. That is surprising as (a) the workforce is aging in most Western countries, (b) many cognitive abilities on average decline with increasing age, and (c) working conditions have been shown to impact the development of cognitive and brain health across the lifespan (e.g., Schooler et al., 1999). The present study aims to fill this gap and investigates work settings which foster positive cognitive and neural plasticity across time.

### The Lifespan Development of Brain and Cognition

Lifespan investigations of cognitive functioning have differentiated between the *mechanics* and the *pragmatics* of cognition (Baltes, 1987; Baltes et al., 2006). *Mechanic* abilities, such as memory, working memory, and processing speed, represent the mostly biology-based processes of the cognitive system which determine the speed and accuracy of elementary information processing. In contrast, *pragmatic* skills are mainly acquired through cultural influences and typically include knowledge-based abilities, such as vocabulary, verbal ability, or general knowledge (Staudinger et al., 1995). Whereas performance levels in pragmatic abilities typically remain stable across the lifespan (or even increase up to late life), the average lifespan development of mechanic abilities is characterized by maturation first (until early adulthood) which then turns into decline. Ample research has provided evidence for this prototypical pattern of adult cognitive development (e.g., Salthouse, 1996; Schaie, 2005).

On a neurophysiological level, aging is associated with fundamental transformations in the structure and function of the brain. Cognitive aging has been associated with an overall volume loss of 14% in gray matter (GM) across the adult lifespan (Jernigan et al., 2001; Kalpouzos et al., 2012) which has mainly been linked to a combination of cell body shrinkage, dendritic regression, and reduced synapse density (Greenwood, 2007; Grady, 2012). Other studies report 0.2–0.5% volume loss per year (Salthouse, 2011). The extent of decline, however, varies by brain region. Frontal and parietal areas seem to be more strongly affected by age than, for instance, the occipital lobe (Hedden and Gabrieli, 2004; Salthouse, 2011). Some research suggests that the striatum (especially the caudate) is particularly prone to age-related regression (Raz et al., 2005; Walhovd et al., 2011). There is evidence that GM volume begins to decline after young adulthood (Greenwood, 2007). Although the available literature is far from conclusive (Raz and Rodrigue, 2006; Salthouse, 2011), there is ample evidence to indicate that higher levels of executive functioning are linked with larger GM volume in the prefrontal cortex (Raz et al., 1998), hippocampal shrinkage mediates age differences in episodic memory (Head et al., 2008), and speed of processing is positively correlated with GM volume in several frontal, parietal, and occipital regions (Chee et al., 2009; see Salthouse, 2011, for a review).

### The Plasticity of Cognitive and Brain Aging

Notwithstanding these age-related changes, the human brain bears potential for *plasticity*, that is, for structural and functional modifications in response to cognitive stimulation (Baltes et al., 2006; Draganski and May, 2008). Lifespan research assumes that such plasticity of brain and cognition is tapped to the extent that an individual is confronted with a mismatch between the functional supply of the cognitive system and challenging environmental demands (Lövdén et al., 2010). The work environment constitutes one kind of environmental demand. The accumulation of such demands across time contributes to the range and limits of the cognitive and brain trajectory (Staudinger et al., 1995). Ample research has provided evidence that stimulating environments are associated with positive *cognitive plasticity* (i.e., the modification of cognitive performance as compared to average levels of cognition at a given age) and *neural plasticity* (i.e., functional or structural changes that occur in the central nervous system as a consequence of mental stimulation). Typical examples include higher levels of cognitive functioning in memory, working memory, and processing speed as well as greater maintenance of GM volume in frontal, parietal, and temporal regions (Valenzuela et al., 2008; Hertzog et al., 2009; Voelcker-Rehage and Niemann, 2013).

It is noteworthy that most of this research has been confined to laboratory settings. ‘*In vivo*’ studies of mental stimulation are few and are often not well controlled (Oltmanns and Staudinger, in preparation). Job complexity is the exception here, and has repeatedly been found to buffer cognitive decline (Schooler et al., 1999). As work takes up a large percentage of full-time employees’ waking hours, work demands can be expected to have a strong influence on cognitive and brain aging.

### Work as an Important Context of Cognitive and Brain Aging

High job complexity refers to work that requires thought and independent judgment including decision-making under ambiguous or contradictory contingencies (Schooler et al., 1999). In contrast, low job complexity is characterized by constrained decision latitude, repetitive work routines, and high standardization (also see Kohn and Schooler, 1978). Cumulative exposure to high job complexity has been found to be associated with better cognitive performance and a lower risk of dementia later in life (Schooler et al., 1999; Potter et al., 2007). Schooler et al. (1999, 2004) found that although individuals with higher baseline cognitive functioning were more likely to be given high complexity jobs, the reciprocal effect of complexity on general cognitive functioning was still present and even somewhat stronger. A recent study corroborated these results and reported positive effects of job complexity on episodic memory (e.g., Fisher et al., 2014). Other groups extended the research on job complexity and took a closer look at the specific effects of complexity with data, people and things (Finkel et al., 2009). Controlling for educational background and using prospective data over 16 years, it depicted that complexity with people was related to changes in speed and visuospatial ability but not to changes in memory. In addition, complexity with data was found to be associated with changes in spatial ability (but not memory and speed). Complexity with things was not related to any cognitive domain.

In a similar vein, recent neurophysiological work has demonstrated that extended exposure to high job complexity (supervisory experience) in midlife was associated with reduced hippocampal GM loss in old age (Suo et al., 2012). Conversely, low levels of job complexity seem to have detrimental effects on cognitive and brain aging: old assembly line workers showed lower task-switching performance and reduced electrophysiological brain activity than a matched control group working in more complex settings (Gajewski et al., 2010).

### Extracting the Effect of Novelty at Work – The Present Study

High job complexity is protective. Jobs with high complexity, however, require higher levels of education. Further, the studies on job complexity have not yet clarified which specific mental challenges are inducing which changes in the cognitive system and whether such changes in principle could also be observed at lower levels of education. The present study hypothesized that recurrent experience of novelty at work (as opposed to over-routinization) induces such changes, and assumes that novelty can be experienced at all levels of complexity (Bowen et al., 2011; Park et al., 2014; Oltmanns and Staudinger, in preparation). Behavioral experiments as well as neurophysiological laboratory studies have established the positive effect of novelty, learning, and skill acquisition (e.g., juggling or learning to decipher Morse code) on cognitive functioning and regional GM volume (Ackerman, 1988; Doyon and Benali, 2005; Draganski and May, 2008; Seidler, 2010; Thomas and Baker, 2013). In contrast, “routinization” has been found to be negatively associated with cognitive flexibility (Tournier et al., 2012), and linked with more pronounced cognitive decline (Bergua et al., 2006; Gajewski et al., 2010).

In a field study using a case-control design, we investigated whether repeated exposure to novelty at work, at low levels of complexity (i.e., under constrained decision latitude, repetitive work routines, and high standardization) was associated with differences in cognitive performance and brain structure in full-time production workers. We focused on one distinct job characteristic, and that is the degree to which (or the lack thereof) a worker had recurrently been confronted with new work tasks that required mastery (i.e., internalization and automatization). Specifically, we compared full-time production workers who experienced multiple (i.e., two or more) versus 0 or 1 work-task changes (WTC) across the 17 years prior to this study in terms of their cognitive functioning and their brain anatomy.

To clarify, WTC refer to changes in work function, that is, intra-organizational changes of the work task, excluding promotion and demotion. Each change implies that one must learn new skill components or ways of dealing with new materials. WTC un-confound the amount of cumulative cognitive challenge through recurrent novelty at work and the amount of cognitive challenge through upward/downward mobility in the sense of more responsibility and decisional latitude. WTC are different from standard forms of job rotation which refers to (short-term) switching between two or more familiar tasks in a fixed temporal sequence (which amounts to repetition; Campion et al., 1994). Whereas multiple WTC (as opposed to prolonged work-task routine) imply repeated acquisition of skill components and the repeated need to learn how to deal with new materials across longer periods of the work life. Multiple WTC therefore may be considered as an ‘*in vivo*’ intervention to stimulate the cognitive system at work.

#### Processing Speed and Working Memory Performance as Leading Indicators of WTC and Cognitive Aging

Recurrent acquisition of new skill components requires individuals to understand and reproduce novel tasks and their specific characteristics (Ackerman, 1988). This is associated with the ability to temporarily store and update new information in working memory. In addition, procedures need to be automatized and streamlined in order to improve performance speed and accuracy which has been related with speed of processing. Therefore, we hypothesized that recurrent learning experiences, repeated acquisition of new skill components, and automation of new work procedures under conditions of multiple WTC as opposed to prolonged routine in 0 or only 1 WTC may have a training effect particularly on processing speed (*H1*) and working memory performance (*H2*).

On a neurophysiological level, skill acquisition is mediated by the ‘cortico-striatal system,’ a network of striatal, frontal, and motor cortical regions (Doyon and Benali, 2005). In line with this, frontal and bilateral striatal activation was, for instance, found in the early stages of motor skill acquisition (Seidler, 2010). Higher demands on the cortico-striatal system in workers with multiple WTC may spark neural scaffolding processes (Reuter-Lorenz and Park, 2014). At the same time, over-routinization and ‘disuse’ of the cortico-striatal system in workers with 0 or 1 WTC may aggravate age-related cognitive decline through reductions in neural activity and decreases in synapse numbers (Valenzuela et al., 2008; Grady, 2012). Thus, we expected multiple WTC to be associated with more GM volume in frontal (*H3*) and striatal regions (*H4*) as revealed by MR imaging. There is abundant evidence which has shown that experiencing control over events in one’s life is a crucial moderator when it comes to determine the outcome of a given event (e.g., Rotter, 1966). We therefore controlled whether such WTC had been reported as voluntary or not. Based on a strong contextual exposure hypothesis, we assumed that the voluntariness would not moderate the effect of novelty processing on cognition and brain structures (*H5*). In addition, we controlled for mental stimulation during leisure time in addition to mental stimulation at work. Even though work occupies a large amount of our waking hours during the week, there is plenty of free time that also exerts influences on cognitive functioning (Andel et al., 2015).

### The Present Study

Our study was conducted with workers of *one* production company. We used a case-control study design to investigate the effect of cumulative exposure to multiple as compared to 0 or 1 WTC on cognitive abilities and brain structure of production workers with differing work biographies (also see Oltmanns et al., 2016). The experimental group consisted of production workers who experienced two or more WTC within the 17 years prior to participation in our quasi-longitudinal study. The control consisted of production workers who experienced 0 or 1 WTC. In order to control for selection biases, we applied a diligent matching procedure on a large number of baseline variables likely to affect the outcome measures.

First, to avoid confounding influences of changes in the job hierarchy (Schooler et al., 1999) or influences of the work environment (e.g., company culture, working rules, etc.), all participants were production workers who worked for the same company at the assembly line or similar monotonous work places without interruption at least between 1996 and 2013. The time window of 17 years prior to participation in our study was chosen because (1) in 1996 the company implemented major changes in its work organization, which we did not want to confound the results, and (2) we needed to identify a time window which was long enough to study the cumulative effect of multiple WTC and which at the same time provided a reasonable number of potential participants (since all participants needed to have stayed with the company at least for the time under study). We wanted to use a treatment window that was as long as it could possibly be to test cumulative effects. The lower boundary, however, was defined by 1996 changes in work organization.

Second, we identified the following variables as important matching variables: age, job type (skilled versus unskilled production work), baseline cognitive functioning (see below), leisure-time activities (current and at baseline, see below), and baseline openness to experience (see below). Current/baseline leisure-time activities need to be taken into account in order to control for cognitive stimulation outside of the work setting. Furthermore, seeking out or accepting task changes may be related to personality as indexed by openness to new experiences (hereafter: openness; McCrae, 1996). Openness goes hand in hand with behavioral flexibility and has also been found to be positively related with cognitive functioning (e.g., Gignac, 2005). To control for the influence of these variables, closely matched pairs of participants were identified according to the following procedure.

## Materials and Methods

### Sampling and Design/Matching

With consent of the works council, the Human Resources Department of the German company identified 3,500 production workers that had been continuously full-time employees in jobs at low levels of job complexity (no promotion or demotion) during the time window of 17 years prior to this study. Afterward, the company distributed a screening questionnaire via the internal mail system that was used to assess all relevant matching variables. Ten female and 166 male workers returned the screening questionnaire (response rate = approximately 5%). On the basis of this information, respondents were organized into subgroups of gender (female/ male), job type (skilled/unskilled production work), and age (30–34, 35–39, 40–44, 45–49, 50–54, 55–59 and more than 60 years of age). Subsequently, we called all 176 potential participants and led semi-structured telephone interviews in order to retrieve the specifics of their work biographies and determine the number of WTC between 1996 and 2013. Participants with two or more WTC versus 0 or 1 WTC between 1996 and 2013 were matched on their baseline cognitive functioning as well as engagement in cognitively stimulating leisure activities and openness at baseline. We classified all participants with 0 or 1 work-task changes since 1996 as ‘0 or 1 WTC’ and all with two and more task changes since 1996 as ‘multiple’ WTC participants.

In order to identify matched pairs, differences of half a standard deviation were used as a range of tolerance around individual scores. Due to a very small number of female workers that satisfied our original selection criteria, our final study sample included only male participants. Out of the remaining 166 male workers, our multi-step matching procedure identified 19 ‘statistical twins’ who were matched in terms of age, educational background, work biographies, indicators of cognitive performance, leisure-time activity as well as openness. **Table 1** indicates that the pairs only differed significantly in the number of WTC (*N* = 38). We purposefully sacrificed a bigger sample size for the sake of minimizing selection biases, and approximating ‘randomization’ in this quasi-experimental design. The final sample of 38 participants was highly selective. After matching, all 38 participants were invited to take part in the next phase of the study which comprised cognitive testing and MR imaging.

**Table 1:** Characteristics of the matched sample (behavioral study).

Matching variables	Work-task changes	
	
	0 or 1 (*N* = 19)	Multiple (*N* = 19)
Age	46.95 (4.38)	46.58 (4.62)
Years of education	12.6 (0.82)	12.30 (1.34)
Gender (Number of male participants)	19	19
Job type (% of unskilled work)	74%	74%
Grade point average (high school; obj. record)	2.99 (0.40)	2.98 (0.42)
LEQ (*z*-scores, young adulthood, reconstructed)	-0.0087 (0.97)	0.0087 (1.06)
Openness (young adulthood, reconstructed)	3.32 (0.49)	3.38 (0.46)
*Task changes*	*0.74 (0.45)*	*3.63 (1.46)*


*Standard deviations are in parentheses. LEQ, Lifetime Experience Questionnaire. Significant group differences at *p* < 0.05 are in italics.*

### Specific Sample for MR Imaging

For security reasons, not all of the 38 participants were eligible to take part in the MRI anatomical brain scanning. Due to magnetic implants, tattoos or claustrophobic tendencies of one or both members, 9 of the 19 pairs of matched participants had to be excluded from the MRI procedure. Only 20 participants (i.e., 10 matched pairs) went through the full experimental procedure including behavioral as well as MRI testing. Thus, all brain anatomical analyses reported in the following are based on 10 pairs of participants (*N* = 20) who were optimally matched on all covariates. **Table 2** provides a description of the reduced MRI sample.

**Table 2:** Characteristics of the matched MRI sample.

Matching variables	Work-task changes	
	
	0 or 1 (*N* = 10)	Multiple (*N* = 10)
Age	46.9 (4.22)	46.8 (4.39)
Years of education	13.0 (0.53)	12.0 (1.87)
Gender (Number of male participants)	10	10
Job complexity (% of unskilled work)	70%	70%
Grade point average (high school; obj. record)	3.01 (0.5)	3.01 (0.49)
LEQ (*z*-scores, young adulthood, reconstructed)	0.0349 (0.94)	-0.0645 (1.18)
Openness (young adulthood, reconstructed)	3.27 (0.54)	3.35 (0.51)
*Task changes*	*0.8 (0.42)*	*3.1 (1.44)*


*MRI, magnetic resonance imaging; LEQ, Lifetime Experience Questionnaire. Standard deviations are in parentheses. Significant group differences at *p* < 0.05 are in italics.*

### Selectivity Analyses

It is important to note that neither the study sample of 38 participants nor the reduced MRI sample of 20 participants differed significantly from the initial 166 male workers who responded to the screening questionnaire in a multivariate ANOVA with age, years of education, academic achievement, health status, work ability, job type, leisure-time activity, and openness in young adulthood as dependent variables. Using Pillai’s trace, the multivariate statistics revealed no group differences, neither between the study sample of 38 participants and the 166 workers who returned the screening questionnaire, *F*(10,155) = 0.92, *p* = 0.50, η^2^ = 0.05, nor between the reduced MRI sample of 20 participants and the 166 workers, *F*(10,155) = 0.72, *p* = 0.70, η^2^ = 0.04.

### Experimental Procedure

Upon arrival all participants were welcomed and received a short overview of the day before they were asked to read and sign the consent form. The entire experimental procedure comprised two parts: part one consisted of a MRI session in which anatomical images were taken of each participant. Part two aimed at collecting the behavioral data. The behavioral part of the study lasted on average 3 h (including two 15-min breaks). Computerized cognitive tests of processing speed and working memory were administered in a controlled laboratory setting. The order of the tasks was randomized for each participant. Other variables that are not part of this study were assessed after the computerized testing procedure. Due to organizational reasons, MRI sessions always had to be scheduled before the behavioral testing. Therefore, participants completed the MRI session, which took 2 h, before starting to work on the behavioral part of the study (after an extended lunch break of 1 h).

### Materials

#### Screening Questionnaire

The screening questionnaire consisted of four parts. Part one gathered information on demographic variables (age, gender, educational attainment, years of education, family status, and number of children). Afterward, a description of each work place held since 1996 was requested. Participants were asked for job title, content, average working hours per week, team size, and employment status. Part three of the screening questionnaire asked for the reconstruction of their engagement in mentally stimulating activities and openness in young adulthood (see below). The fourth and last part asked for the high school grade point average (GPA) as a proxy of baseline cognitive functioning.

##### Approximating leisure-time activity in young adulthood

In order to assess leisure-time activity in young adulthood, we used a translated version of the Lifetime of Experiences Questionnaire (LEQ; e.g., Valenzuela and Sachdev, 2007). The LEQ determines a person’s cognitive stimulation through education, complex occupations, and cognitively stimulating leisure activities across the lifespan. It comprises 42 items and is subdivided into age-specific and non-specific parts. Since we already had the information about education and occupational history, we excluded these items but used the remaining questions to assess former (in young adulthood) and current participation in a broad range of mentally stimulating leisure-time activities (e.g., reading, writing, giving lectures, playing a music instrument, learning a second language or being engaged in physical activity). The LEQ is a highly reliable instrument (test–retest reliability: *r* = 0.98) which was shown to be a valid predictor of longitudinal cognitive change as well as brain atrophy (Valenzuela et al., 2008).

##### Approximating Openness in young adulthood

To approximate baseline openness, nine items were created on the basis of the German version of the Big Five Inventory (Rammstedt and John, 2005). In order to minimize recall biases and to avoid ‘telescoping effects’ (Rubin and Baddeley, 1989), we created items that asked to recall concrete behaviors linked with salient and noteworthy life events. To further improve retrieval, we asked them to recall specific living conditions and significant life events during that period of their life (to set landmarks and create a temporal reference system; Friedenreich, 1994). A 5-point Likert scale (ranging from 1, *completely disagree* to 5, *completely agree)* was used to assess agreement with statements like “*When I was 16 to 25 years old, I attended many different music events*” or “*When I was 16 to 25 years old, I traveled a lot.*” The average of these nine items constituted a scale for (reconstructed) openness in young adulthood (α = 0.68). Bivariate correlation of this baseline openness measure with the openness scale of the German BFI version was highly significant and very satisfactory (*r* = 0.64). It also correlated moderately high with a scale of flexible goal adjustment (*r* = 0.45) (Brandtstädter and Renner, 1990) as well as the LEQ scale for mental activity in young adulthood (*r* = 0.26).

##### Approximating cognitive performance in young adulthood

As we cannot rule out the possibility that cognitively more able individuals are prone to experience more WTC (although there seems to be no relationship between cognitive ability and at least job mobility; see Griffeth et al., 2000), we wanted to control for level of cognitive performance at baseline. We used the high-school GPA as proxy variable. It is widely accepted that cognitive ability is associated with academic achievement. Ample research has established correlations of about 0.4 to 0.7 between IQ scores and school performance (Deary et al., 2007). In addition, a recent meta-analysis found that out of 112 studies that assessed the relationship between personality traits and intellect, 86 utilized academic achievement as proxy variable of intellect. Next to intelligence tests, it was the most often employed indicator of cognitive functioning (von Stumm and Ackerman, 2013). Therefore, we used the high school GPA as a proxy for baseline cognitive functioning. It was calculated as a mean of six grades retrieved from the graduation certificates of each participant: German, English, mathematics, physics (if not available: chemistry), history (if not available: geography) and arts. Bivariate correlations of these averages with the cognitive performance measures used below were satisfactory (*r* = 0.30–0.43) and comparable to long-term correlations between grades and cognitive performance reported in the literature (Deary et al., 2007).

##### Reconstruction of work-task biographies

Individual work-task biographies were assessed via telephone. Four trained interviewers led semi-structured biographical interviews. Each interview started with the question “*Which tasks and duties does your current position include? Please give a detailed description.*” Afterward, participants were asked “*Were there ever any changes in your tasks and duties since 1996? If yes, when?*” Subsequently, the interviewer went through the work-task biography in reverse. Starting with the present job tasks, each position within the last 17 years was discussed step-by-step and comprehensively documented by the interviewer. Every interviewee was required to give a detailed description of all tasks since 1996 (e.g., engine assembly or interior fittings). This diligent step-by-step procedure served to reduce recall biases and helped to draw a complete picture of the work biographies of our participants. Also, note that for the present design the absolute number of WTC was of less relevance than the basic distinction between ‘0 or 1’ or ‘multiple.’ In addition, since the company implemented an extensive change in work organization in 1996, the recollection of task allocation in this ‘anchor’ year allowed reducing recall biases. Afterward, ‘multiple’ and ‘0 or 1’ WTC were determined for each participant in consensus meetings that involved all four interviewers as well as the project members. To further test the reliability of our WTC measurement, we assessed the number of WTC twice: first, in the telephone interview prior to the lab study and second during the lab study itself. There was not one participant reporting conflicting information on both occasions.

### Cognitive Tests

As processing speed and working memory capacity have been linked with skill acquisition and learning, we included both cognitive measures.

#### Processing Speed

Processing speed was measured via two tasks: the visual search task (Hommel et al., 2004) and the identical pictures test (Ghisletta et al., 2006). It was important to us to select processing speed tasks with high external validity. We expected the visual search task and the identical pictures test to be more closely linked with the day to day tasks of the participating production workers than other speed measures. The *visual search task* is often used as a standard test for processing speed and has been shown to be sensitive to age effects (Foster et al., 1995; Hommel et al., 2004). The version we employed in our study was similar to that used by Voelcker-Rehage et al. (2011), apart from the fact that we exclusively used conjunction searches with a set size of 14 stimuli. Participants had to search a target (filled white circle) among 14 unfilled circles and filled white squares and were instructed to press a left button with their left index finger if they found the target and press a right button with their right index finger in case they did not find it. It was emphasized to respond as quickly and as accurately as possible. In total, participants had to work on five blocks with 80 trials each (50% target present trials). They were not given any practice run but received standardized instructions and illustrated examples. *Z*-scores of the median reaction times of correct trials and the response accuracy were used to create a composite score of the visual search task as outcome variable.

In the *identical pictures test* a target figure was presented in the upper half and five response alternatives were presented in the lower half of a computer screen. Participants were instructed to identify as fast and as accurately as possible the one figure among the five response alternatives that equals the target figure and click on it (Ghisletta et al., 2006). Each trial ended at first response and was then followed by the next. In total, 46 trials were available. However, the test ended automatically after 80 s. According to the literature (see Ghisletta et al., 2006), the identical pictures test uses the number of correctly solved trials within this time frame as a measure of processing speed. Therefore, *z*-scores of the number of correctly solved trials within 80 s were used in the statistical analyses. Reliability of the identical pictures test was very satisfactory (Cronbach’s alpha = 0.80–0.96).

We used both of these rather dissimilar measures because they target different levels of difficulty. Whereas in the visual search task participants had to compare simple geometric shapes, the identical pictures test used more complex figures. The identical pictures test forced participants much more than the visual search task to pay attention to details. In line with this rationale, visual search theory suggests that higher difficulty is more sensitive in carving out existing dissimilarities in cognitive functioning (Wolfe, 2007). That is, lower levels of difficulty in the visual search task may mitigate existing differences in cognitive functioning between participants with multiple versus 0 or 1 WTC whereas higher levels of intricacy in the identical pictures test may aggravate such dissimilarities. See **Table 3** for the 0-order correlations between the matching variables and the cognitive variables.

**Table 3:** Zero-order correlations of the matching variables with the cognitive variables.

	Matching variables						Cognitive variables		
		
	Age	Years of education	Job complexity	GPA	LEQ (rec.)	Openness (rec.)	Visual search	Id. Pic.	N-back
Age	1								
Years of education	-0.40*	1							
Job complexity	0.07	0.05	1						
GPA	0.26	0.19	0.04	1					
LEQ (rec.)	-0.16	0.25	-0.07	-0.30^†^	1				
Openness (recon.)	0.03	0.08	0.37*	-0.26	0.35^∗^	1			
Visual search	-0.33*	0.00	0.23	-0.42^∗^	0.40^∗^	0.25	1		
Identical pictures	-0.27^†^	-0.07	0.19	-0.29^†^	0.15	0.17	0.52^∗^	1	
N-back	0.00	0.00	0.31^†^	0.00	0.11	0.04	0.23	0.32^∗^	1


*GPA, grade point average; LEQ (rec.), Lifetime of Experience Questionnaire (reconstructed from young adulthood); Openness (recon.), openness to experience (reconstructed from young adulthood); Id. Pic., identical pictures; ^∗^*p* < 0.05; ^†^*p* < 0.1.*

#### Working Memory

Working memory performance was assessed with the N-back task (Jaeggi et al., 2010). Participants had to remember a span of individually presented letters and compare the current item with the one before. The task was administered at two levels of difficulty, as visual 1-back and 2-back task. The visual 1-back and 2-back tasks are reliable measures to asses working memory capacity (split-half reliability: *r* = 0.94) with satisfactory psychometric validity (Jaeggi et al., 2010). In the 1-back task, participants were told to press a left button with their left index finger whenever the current letter equaled the one presented immediately before. If the current letter was different, they were instructed to press a right button with their right index finger (Voelcker-Rehage et al., 2011). Similarly, in the 2-back task, they had to press the left button if the current letter was equal to the one presented two items earlier, and press the right button if this was not the case. All subjects started with the 1-back task and were shown a randomized sequence of 80 letters at both levels of difficulty (37.5% match trials). Each letter was presented for approximately 1500 ms and was then followed by a fixation cross (exposure time = 300 ms). As in the visual search task, participants were not given practice trials. They received a standardized instruction and digitally illustrated examples in which it was emphasized to respond as accurately as possible. *Z*-scores of the response accuracies of the 1-back and 2-back tasks were used to create a composite N-back score as outcome variable.

### Control Variables

#### Leisure-Time Activity

As stated above we used a translated version of the LEQ (Valenzuela and Sachdev, 2007) to assess former (in young adulthood) as well as current participation in a broad range of mentally stimulating leisure-time activities. *Z*-Scores of the sum of current participation in stimulating leisure-time activities (as indicated by LEQ items) were used as control variable.

#### Voluntariness of WTC

In order to control for the potential influence of voluntariness of WTC, we additionally assessed voluntariness of each WTC over the 17-year period. Using a 5-point Likert scale (ranging from 1, *absolutely voluntary* to 5, *absolutely not voluntary)*, participants were asked “*Was this a voluntary work-task change?*” for each WTC. The mean voluntariness across all WTC over the 17-year period was used to indicate voluntariness in WTC.

### MR Data Acquisition and Analysis

#### Acquisition

Voxel-based morphometry (VBM) was applied to T1-weighted anatomical brain scans acquired on a 3-Tesla Siemens Allegra whole-body magnetic resonance tomograph (MPRAGE sequence, TR of 2300 ms, 176 slices with 1 mm × 1 mm × 1 mm isotropic resolution). The anatomical brain scans were part of a larger MRI protocol. The entire MRI protocol lasted about 90 min.

Preprocessing and analysis of T1-weighted images were performed using the VBM 8 toolbox^1^ (Structural Brain Mapping Group, University of Jena, Germany) in SPM8^2^ (Wellcome Trust Centre for Neuroimaging, University College London, London, UK) running on MATLAB version R2011b (The MathWorks, Sherborn, MA, USA). We applied the standard VBM8 routines and default parameters. The preprocessing procedure implemented in VBM8 consists of (1) a correction for bias-field in homogeneity, (2) a high-dimensional spatial DARTEL (Diffeomorphic Anatomical Registration Through Exponentiated Lie Algebra) normalization into MNI (Montreal Neurological Institute) space, (3) tissue segmentation into GM, white matter (WM), and cerebrospinal fluid (CSF), and (4) a modulation step in which GM images were multiplied by the local value derived from the deformation field (in order to account for individual brain size differences and restore within-voxel volumes that may have been altered during normalization). The modulated GM volumes were smoothed with an 8 mm FWHM (full width half maximum) Gaussian kernel. The normalized, modulated, and smoothed GM images were used for statistical analyses.

#### Statistical Analysis

Comparisons of regional GM volumes between participants with multiple versus 0 or 1 WTC in 17 years were performed using both voxel- and cluster-level inference within the framework of the general linear model. Full-factorial ANCOVA was used to investigate regional GM volume differences across the whole brain between the two groups. Prior to analysis, GM volumes with less than 0.2 tissue class probability were excluded. Due to the small sample size, only current participation in cognitively stimulating leisure-time activities (but not voluntariness in WTC) was added to the model as covariate. Statistical parametric GM maps were thresholded with *p* < 0.001 (uncorrected). We used the empirically determined extent threshold of *k* = 56 voxels per cluster to correct for multiple comparisons. That is, only voxel clusters exceeding a size of more than 56 voxels will be reported. A recent study provided evidence that reporting uncorrected results from parametric statistical approaches tends to inflate false-positive error rates (Eklund et al., 2016). However, the same study also reported that cluster-defining thresholds of *p* < 0.001 combined with a smoothing kernel of 8 mm yields expected levels of false-positive error rates in SPM. In addition to that, we are optimistic that our extent threshold of *k* = 56 voxels helps to minimize potential errors.

#### Correlating Individual Brain Volume with Cognitive Performance

We extracted individual GM volumes from significant VBM clusters with the help of the MarsBar toolbox for SPM^3^, and related the average GM volume in each cluster with performance in the significant cognitive tests (in a partial correlation analysis, controlling for current leisure time activity).

## Results

### Are Work-Task Changes Associated with Higher Levels of Cognitive Performance?

Due to the rather small sample size, the behavioral hypotheses were tested via one Multivariate Analysis of Covariance (MANCOVA) - rather than using multilevel modeling – with (a) a composite score for the visual search task built from averaged *z*-scores of the median reaction time per correct trial and from *z*-scores of the accuracy in the visual search task, (b) *z*-scores of the number of correct trials within 80 s in the identical pictures test, and (c) a composite *z*-score of the accuracies in the 1- and 2-back task as dependent variables. WTC (‘multiple’ versus ‘0 or 1’) were included as independent variable. A composite LEQ score and its interaction with WTC as well as an average *z*-score of voluntariness in WTC served as covariates. The results for the main effects of WTC after controlling for leisure-time activity and voluntariness in WTC can be found in **Table 4**.

**Table 4:** Differences in cognitive performance as a function of work-task changes (adjusted for leisure-time activity and voluntariness; *n* = 38).

Indicators of cognitive performance	Work-task changes			
	
	0 or 1 (*N* = 19)	Multiple (*N* = 19)	*F*	*P*
Visual search	-0.89 (0.19)	0.13 (0.19)	1.16	0.29
Identical pictures	-0.12 (0.21)	0.12 (0.21)	6.81	0.01
N-back	-0.18 (0.16)	0.25 (0.16)	5.63	0.02


*Indicators of cognitive performance are presented in composite *z*-scores. Standard errors are given in parentheses.*

Using Pillai’s trace, the multivariate statistics revealed a main effect of WTC on cognitive functioning, *F*(3,31) = 3.32, *p* = 0.03, η^2^ = 0.24, as well as a marginally significant interaction between WTC and leisure time activity, *F*(3,31) = 2.79, *p* = 0.06, η^2^ = 0.21, but no main effect of leisure-time activity, *F*(3,31) = 1.48, *p* = 0.24, η^2^ = 0.13, and voluntariness in WTC, *F*(3,31) = 2.03, *p* = 0.13, η^2^ = 0.16. Given the significant omnibus effect, we decided to accept this pattern of results as indication of a potential main effect of WTC and inspected the univariate statistics for processing speed and working memory performance.

#### Processing Speed

**FIGURE 1:**
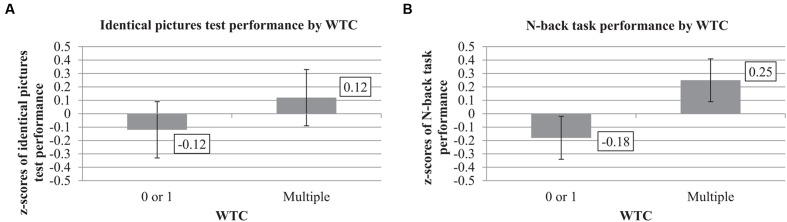
**Identical pictures test and N-back performance by work-task changes (WTC) (adjusted for leisure-time activity and voluntariness in WTC; *n* = 38).** *z*-scores of performance in the identical pictures test **(A)**, and N-back task **(B)** by multiple versus 0 or 1 WTC during 17 years. Error bars indicate standard errors.

**FIGURE 2:**
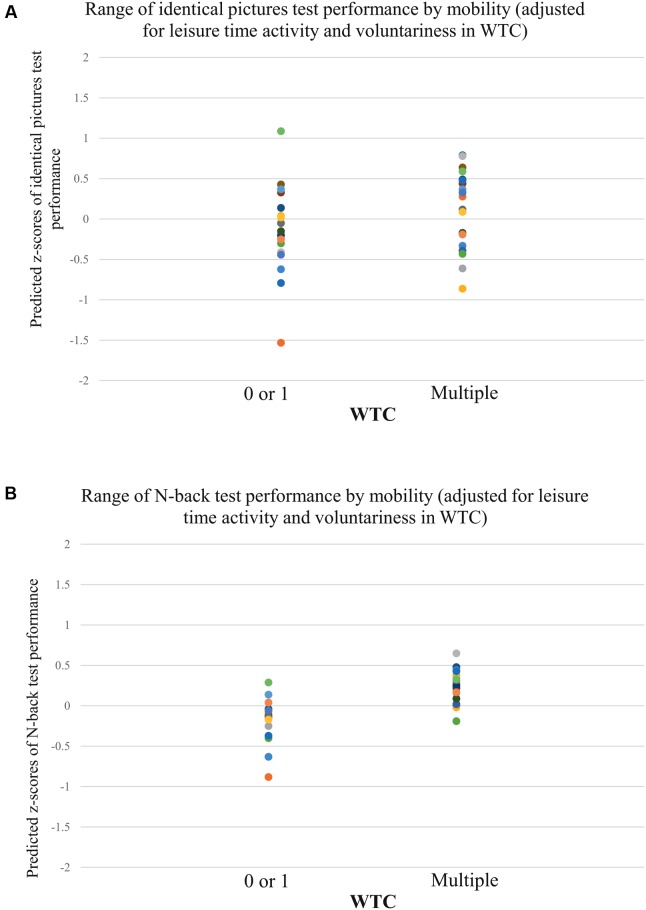
**Individual test performance in identical pictures test and N-back task by WTC (adjusted for leisure-time activity and voluntariness in WTC; *n* = 38).** Individual *z*-scores of performance in the identical pictures test **(A)**, and N-back task **(B)** by multiple versus 0 or 1 WTC during 17 years.

The univariate statistics showed a significant effect of WTC on the identical pictures test, *F*(1,33) = 6.81, *p* = 0.01, $\eta_{p}^{2}$ = 0.17. Participants with multiple WTC reached higher *z*-scores in the identical pictures test (*M*_multiple_ = 0.12, *SE* = 0.21) than participants with 0 or 1 WTC (*M*_0or1_ = -0.12, *SE* = 0.21). See **Table 4** and **Figures 1** and **2** for further information.

The univariate statistics revealed no main effect of WTC on performance in the visual search task, *F*(1,33) = 1.16, *p* = 0.29, $\eta_{p}^{2}$ = 0.03. See **Table 4**. We interpreted this finding as a potential ceiling effect as a consequence of lower cognitive load as compared to the identical pictures test. The average reaction time across all participants and conditions of the visual search task (*M*_all_ = 675.6 ms) was more than 200 ms lower than in a comparable subsample of the lifespan study by Hommel et al. (2004; *M*_Hommel,45-55 years_ = 876 ms). In addition, the standard deviation was only a fourth of that presented in Hommel’s work (*SD*_all_ = 65.1 ms versus *SD*_Hommel,45-55 years_ = 253 ms).

#### Working Memory

**Table 4** summarizes the significant univariate effect of WTC on the N-back task, *F*(1,33) = 5.63, *p* = 0.02, $\eta_{p}^{2}$ = 0.15. As shown in **Figures 1** and **2**, multiple WTC led to higher composite *z*-scores in the N-back task than 0 or 1 WTC (*M*_multiple_ = 0.25, *SE* = 0.16 vs. *M*_0or1_ = -0.18, *SE* = 0.16).

### Are Work-Task Changes Associated with Differences in Brain Structure?

**FIGURE 3:**
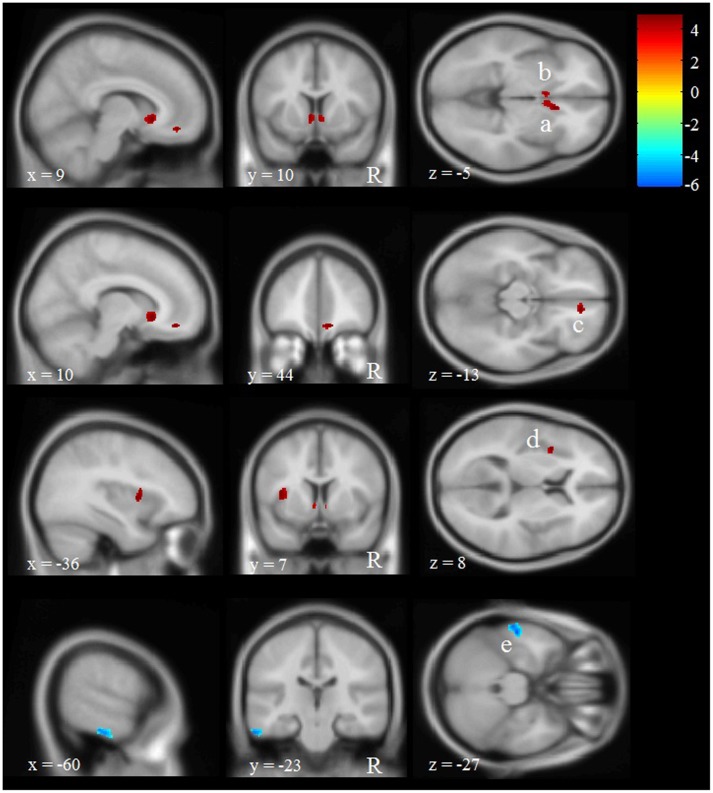
**Effects of WTC on regional gray matter (GM) volume in long-term production workers (*n* = 20).** Shown are clusters with significantly more (red, a–d) or less (blue, e) GM volume in participants with multiple WTC as opposed to participants with 0 or 1 WTC. Multiple WTC were associated with more GM volume in the left (a) and right caudate (b), as well as in the medial frontal gyrus (c) and the insular cortex (d). 0 or 1 WTC were associated with more GM volume in the inferior temporal gyrus (e). The letter ‘R’ indicates the right hemisphere. Letters a, b, c, d, e refer to the voxel clusters depicted in **Table 1**. x, y, and z specify the MNI coordinates.

The VBM analysis revealed five regions with significant differences in GM volume between participants with multiple versus 0 or 1 WTC (see **Figure 3** and **Table 5**). We found four voxel clusters in which participants with multiple WTC indicated more GM volume than participants with 0 or 1 WTC: two clusters located in the left and right caudate, one of them extending into the right rostral anterior cingulate cortex (ACC). In addition, there were another two clusters in the right medial frontal gyrus and in the left insula. Vice versa, participants with 0 or 1 WTC depicted more GM volume in the left inferior temporal gyrus.

**Table 5:** Differences in regional gray matter (GM) volume between production workers with multiple versus 0 or 1 WTC (*n* = 20).

Contrast: multiple WTC > 0 or 1 WTC							

Region	Hemis-phere	Letter Figure 2	MNI coordinates			Cluster size	*t*-value (df = 15)
					
			*x*	*y*	*z*		
Caudate/ACC	R	A	11	21	-6	195	4.91
Caudate	L	B	-5	11	-3	62	4.47
Medial frontal gyrus	R	C	11	44	-14	67	4.84
Insula	L	D	-36	6	4	109	4.86
**Contrast: 0 or 1 WTC > Multiple WTC**							
Inferior temporal gyrus	L	E	-56	-21	-29	267	5.95


*WTC, work-task changes; Fig., Figure; MNI, Montreal Neurological Institute; R, right; L, left. ox*t*-values at *p* < 0.001 (uncorrected). Extent threshold (*k*) = 15 voxels.*

In a follow-up analysis and in order to validate the factual importance of the regions detected in our VBM analysis, we correlated the individual average GM volume in each of these regions with performance in the identical pictures and N-back tests. Results suggest moderately high correlations between performance in the identical pictures test (but not the N-back task) and the four clusters that were positively associated with WTC: particularly the two clusters comprising the left and right caudate and rostral ACC but also the two clusters in the left insula and the right medial frontal gyrus depicted moderately high correlations between *r* = 0.22 and *r* = 0.46 with performance in the identical pictures test. That is, the more GM volume individuals depict particularly in the left and right caudate the more correct trials they reached in the identical pictures test. In contrast, the one cluster in the left inferior temporal gyrus that was negatively related to WTC did not show a significant correlation with processing speed or working memory (see **Table 6**).

**Table 6:** Correlations of average GM volumes with cognitive performance (*n* = 20).

Brain areas	Hemis-phere	Letter Figure 2	Indicator of cognitive performance	
			
			Identical pictures	N-back
Caudate/ACC	R	a	0.403^†^	-0.034
Caudate	L	b	0.466^∗^	-0.032
Medial frontal gyrus	R	c	0.227	0.113
Insula	L	d	0.346	-0.144
Inferior temporal gyrus	L	e	-0.126	-0.233


*Average GM volumes were partially correlated with composite *z*-scores of performance in the identical pictures test and N-back task. VBM, voxel-based morphometry; R, right; L, left; ^∗^*p* < 0.05, ^†^*p* < 0.1.*

## Discussion

All in all, our results supported and extended our hypotheses. We provided first cautious evidence that recurrent experience of novelty at work (as indicated by multiple WTC in 17 years) is associated with (a) higher levels of processing speed and working memory performance and (b) greater GM volume in striatal, frontal, and insular regions. This is first evidence that novelty seems to play a role in counteracting the debilitating effects of low complexity at work. However, replication is needed to corroborate these results.

### Mental Stimulation through Novelty

#### Cognitive Performance

Our findings are consistent with the interpretation that a work environment which is characterized by multiple (as opposed to 0 or only 1) WTC implies repeated confrontation with new tasks and recurrent skill acquisition. Building on such findings one might argue that cumulative long-term negative effects of low complexity work could be avoided by systematically introducing work-task changes. Our results suggest that recurrent novelty of the work tasks seem to trigger plasticity (even on low levels of complexity and independent of leisure-time behavior and voluntariness in WTC). Thus, it may be considered one crucial component of job complexity, however, it is one that can occur at all levels of complexity. Recurrent novelty (at work or in general) may be one critical contextual feature for cognitive plasticity to unfold. It may be the mismatch that has been postulated in earlier influential work (Lövdén et al., 2010). This is in line with recent evidence suggesting that cognitive and brain plasticity may particularly benefit from active learning and novel information processing (Park et al., 2014).

#### Brain Structure

The behavioral results were replicated in the analyses of potential difference in brain structure. We found that multiple WTC in 17 years were associated with more GM volume in striatal, frontal, and insular regions. In the context of long-term exposure to the detrimental effects of repetitive production work, more as compared to less WTC may have placed higher demands on the cortico-striatal system (Doyon and Benali, 2005; Seidler, 2010) which in turn affected GM volume in parts of the medial frontal gyrus as well as in the left and right caudate. The affected brain regions are also part of the dopamine system (Li et al., 2010). The dopamine system is assumed to play an essential role in rewarded learning processes (Bäckman et al., 2010) and also declines with age, mainly in the caudate but also in frontal regions and the anterior cingulate (see the ‘correlative triad’ between age, dopamine receptor loss and cognitive functioning; Bäckman et al., 2010). It will be interesting to see in future research whether recurrent novelty and learning experience due to multiple WTC as opposed to prolonged routine due to less WTC have the potential to diminish age-related receptor loss in these regions.

The mechanisms that underlie the effect of WTC on GM volume in the rostral ACC and the insular cortex may be more difficult to understand. However, both regions depict age-related GM volume loss (Mann et al., 2011; Taki et al., 2013) and both regions seem to be related to learning processes and cognitive functioning. To start with, the ACC is commonly involved in error detection and conflict monitoring (Bush et al., 2000; Elmer et al., 2014). Experimental research has provided evidence for the monitoring role of the ACC in executive functioning (Abutalebi et al., 2012; Elmer et al., 2014). The insular cortex, on the other hand, is involved in a multitude of processes. Next to visceral and autonomic activities such as heart rate, bladder and bowel distension, there is evidence that it plays an important role in cognitive functioning (e.g., Nelson et al., 2010). For instance, the insular cortex was related to working memory performance, processing speed, and executive functioning (Zakzanis et al., 2005; Ruscheweyh et al., 2013; Müller et al., 2014). Indeed, it has been stated that a functional network involving the insular cortex and parts of the ACC and the medial frontal cortex are among the most frequently activated brain regions in any cognitive task (Ebisch et al., 2013). The connectivity strength of this network has been linked to higher levels of performance in tests of executive functioning and logical reasoning (Ebisch et al., 2013; Müller et al., 2014). Interestingly, a recent review postulated the striatum as well as the insular cortex and the medial frontal cortex to be part of a more complex loop to facilitate decision making (cf. the ‘affect-integration-motivation framework’; Samanez-Larkin and Knutson, 2015).

### Limitations and Future Research

Why and how WTC affect cognitive performance is an open question. We suggest that our findings are consistent with the interpretation that multiple as opposed to 0 or 1 WTC during 17 years represent a work context that is characterized by repeated confrontation with new, yet unknown situations and cognitive challenges. And it is especially these characteristics that are essential to maintain (or spark positive changes in) adult cognitive functioning. In this vein, our results may support the notion that instead of the complexity of a given environment it may rather be the novelty that is critical for cognitive plasticity to manifest (Lövdén et al., 2010; Bowen et al., 2011; Park et al., 2014).

Although this study uncovered some interesting results, it does have limitations. With 38 (20) participants in total and 19 (10) participants in each of the two experimental groups, the statistical power of our analyses was low, particularly in the reduced fMRI sample. Therefore, some effects may have failed to reach conventional levels of significance. However, samples of 20 subjects have been shown to yield acceptable levels of false-positive error rates (e.g., Eklund et al., 2016). It is important to note that this study was exploratory field research, conducted, however, under rather controlled conditions. We aimed to minimize selection biases via a complex matching procedure.

‘*In vivo*’ research is time consuming as it is complex to enter real-life environments and establish a quasi-experimental design. It took the project team almost 2 years to develop the trust with the company leadership and its work council in order to finally gain access to human resource information and to workers. Of course, traditional randomization of individuals to work biographies with more or less WTC is not conceivable. Thus, quasi-experimental design with its limitations was the design of choice. A case-control study design represented the most rigorous approach. Therefore, without doubt replication studies are needed to rule out that effects are due to either chance or selection. We would like to present these study results as a first cautious hint as to which components of cumulative work settings might be levers to promote cognitively health aging.

As research within a company across a period of almost 20 years is unheard of, we had no option but to reconstruct most of the baseline level variables. In terms of the brain variables we had no baseline assessment available. As a consequence, we cannot entirely rule out the possibility of selection biases. Our hope, however, is that we minimized this problem with a diligent multi-step matching procedure including all relevant covariates. Furthermore, we took great care to develop retrospective measures that were constructed to approximate the baseline covariates with the best possible validity (e.g., test–retest reliability of the LEQ was *r* = 0.98; internal consistency of our scale for reconstructed Openness α = 0.68). However, we have to acknowledge that these scales remain being reconstructions and we cannot say to what extent our participants were able to accurately recall and report their engagement in mentally stimulating activities and openness in young adulthood.

**FIGURE 4:**
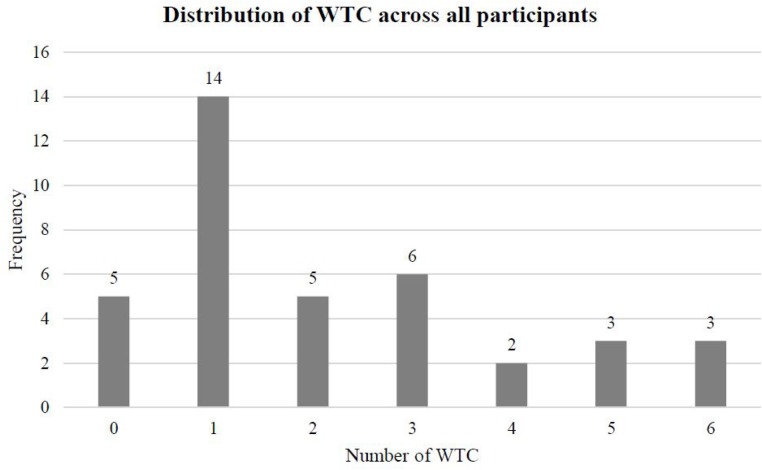
**Distribution of WTC across all participants (*N* = 38)**.

In a similar vein, the number of WTC was assessed retrospectively in biographical interviews. All work biographies were discussed in reverse, starting with the present job tasks. We are convinced this step-by-step procedure served to reduce recall biases and helped to draw a complete picture of the work biographies of our participants (see **Figure 4** for the distribution of WTC across all participants). However, we cannot guarantee the accuracy of our measurement. It is possible that there is a causal relationship between the ability of our participants to accurately remember their work biographies and the dependent variables in this study (namely, working memory, processing speed, and GM volume).

Similarly, we used the GPA as proxy variable for baseline cognitive functioning. Next to intelligence tests, GPA was the most often employed indicator of cognitive functioning (von Stumm and Ackerman, 2013). And correlations of about 0.4–0.7 between IQ scores and school performance (Deary et al., 2007) provide further support for its validity to proxy cognitive functioning. But GPA is likely to be determined by factors other than cognitive functioning as well, such as, motivation, environmental support, social background, or teacher ability. It is possible that we were not able to fully control for selection biases with GPA as measurement for baseline cognitive functioning. We cannot avert the possibility of reverse causality, that is, that participants with higher baseline cognitive functioning were more likely to be given new or other work responsibilities because of their capability. However, to additionally control for this, we added voluntariness in WTC to our analysis model. We did not find any moderator effect voluntariness. Obviously, future studies should aim for prospective longitudinal designs with greater sample sizes and pre- and post-assessment of all relevant covariates and measures of GM volume.

Whether the group differences in cognitive performance and regional GM volume are the positive consequence of more mental stimulation in participants with multiple WTC or the negative consequence of over-routinization in participants with 0 or 1 WTC or both, we cannot determine. On the basis of our data, we cannot disentangle the mechanisms that underlie the promising effect of WTC. For instance, whether the GM volume differences between participants with multiple versus only one or less WTC in 17 years are a consequence of increments in GM or a consequence of reduced loss under multiple WTC, we cannot say. As we interpret our results, repeated confrontation with novel experience, skill acquisition and automatization of new routines placed higher demands on the cortico-striatal (and perhaps the dopaminergic) system. These characteristics may have sparked neural scaffolding processes (e.g., augmented synaptogenesis; see Markham and Greenough, 2004; also see Petersen et al., 1998; Reuter-Lorenz and Park, 2014). At the same time, over-routinization and ‘disuse’ of the cortico-striatal system in workers with less WTC may have aggravated age-related cognitive decline through reductions in neural activity and decreases in synapse numbers (Valenzuela et al., 2008; Grady, 2012). As noted above, it is likely the strong effect size of the differences in cognitive performance and GM volume in participants with more versus less WTC are linked with the increased sensitivity that was built into our design by confining ourselves to one company and by the rigorous matching procedure that we applied. Thus, the variance in outcome variables may be reduced and therefore favor stronger effect sizes. The observed differences between participants with more versus less WTC may therefore be amplified. Apart from this magnifying effect, it is also possible that recurrent experience of novelty (i.e., recurrent WTC) may have triggered new behaviors at work or in private life (over and above the controlled leisure-time activities) which lead to a cascading effect and augmented the group differences. In other words, the positive effects of recurrent novelty at work (e.g., recurrent skill acquisition, better cognitive performance, increase of GM volume in cortico-striatal networks) spilled over to private life and paved the way to more cognitively favorable behavior or environments outside of work which then accumulated over the years and supposedly affected the exposure of novelty at work in turn (in the sense of an upward spiral of mental stimulation at work and in leisure time). This speculation also deserves further investigation in future research.

Another interesting avenue for the studies to come is the long-term effect of WTC. In the present work, we studied middle-aged workers (*M*_age_ = 47 years). It is an open question whether the small effects we found with this comparatively young sample would be more pronounced in an older population. There is reason, however, to assume that a career of multiple versus 0 or 1 WTC could unfold greater influence later in life. For instance, job complexity showed greater effects in older than in younger workers (Schooler et al., 1999). Whether the same holds true for WTC is an interesting question that should be dealt with in future work.

Finally, and regardless of the rather theoretical considerations above, our findings may have important practical implications. Low complexity occupations such as industrial production work have detrimental effects on brain and cognition. However, an average of three to four WTC in 17 years yields considerable differences in both cognitive performance levels and brain anatomy. In other words, one WTC in 4–5 years could already help to preserve cognitive health and facilitate work ability, well-being, and productivity across the working lifespan at low levels of job complexity. Managing directors, company owners, as well as personnel and health executives may therefore want to consider aging as an important variable in organizational health psychology and understand WTC as strategic health management instrument.

## Conclusion

Taken together, the present study demonstrated optimistic evidence that recurrent experience of novelty (as indicated by multiple WTC in 17 years) can serve as a powerful ‘*in vivo*’ cognitive intervention in the work setting to diminish negative long-term effects of low job complexity on the cognitive system (Gajewski et al., 2010). Moreover, our findings may extend extant knowledge on critical contextual features that foster cognitive plasticity: it is possible that recurrent experience of novelty at work is a crucial component underlying the observed effects of job complexity. Future research, however, needs to corroborate our first cautious evidence.

## Ethics Statement

This study was approved by Ethik-Kommission der Deutschen Gesellschaft für Psychologie (DGPs).

## Author Contributions

BG, KS, and US (in alphabetical order) developed the study concept. JO, US, BG, AW, GR, KS, and CV-R contributed to the study design. Testing and data collection were performed by JO, AW, and GR. JO, AW, and CN performed the data analysis and interpretation under the supervision of BG and US. JO, US, and BG drafted the manuscript, and all other authors provided critical revisions.

## Conflict of Interest Statement

The authors declare that the research was conducted in the absence of any commercial or financial relationships that could be construed as a potential conflict of interest.

## Footnotes

1 http://dbm.neuro.uni-jena.de/vbm/

2 http://www.fil.ion.ucl.ac.uk/spm/

3 http://marsbar.sourceforge.net
